# Real-World Effectiveness of RSVpreF and RSVpreF3 Vaccines in Preventing Hospitalization and Emergency Department Visits Associated With Respiratory Syncytial Virus in Older Adults: A Meta-analysis

**DOI:** 10.1093/cid/ciag107

**Published:** 2026-02-18

**Authors:** Dewan Md Sumsuzzman, Congjie Shi, Seyed M Moghadas

**Affiliations:** Agent-Based Modelling Laboratory, Centre of Excellence in AI for Public Health Advancement, York University, Toronto, ON, Canada; Laboratory for Industrial and Applied Mathematics, York University, Toronto, ON, Canada; National Laboratory for Health Security, University of Szeged, Szeged, Hungary; Agent-Based Modelling Laboratory, Centre of Excellence in AI for Public Health Advancement, York University, Toronto, ON, Canada

**Keywords:** respiratory syncytial virus, vaccine effectiveness, older adults

## Abstract

**Background:**

Respiratory syncytial virus (RSV) vaccines were first recommended for older adults during the 2023–2024 season in countries that authorized their use. Although early observational studies complemented trial findings, real-world evidence on vaccine effectiveness against severe RSV disease remains limited. We assessed the effectiveness of RSVpreF and RSVpreF3 vaccines in preventing RSV-related hospitalizations and emergency department (ED) visits among older adults.

**Methods:**

We searched MEDLINE, Embase, Web of Science, Scopus, Global Health, and medRxiv for observational studies published between 1 January 2023 and 30 December 2025, reporting real-world effectiveness of RSV vaccines in adults aged 60 years or older. Pooled analyses used inverse-variance random-effects models to estimate effectiveness against RSV-related hospitalizations and ED visits. Subgroup analyses assessed differences in effectiveness by age, immune status, and vaccine type. This study is registered with PROSPERO (CRD420251021777).

**Results:**

From 4925 records screened, 8 cohort and case-control studies were included. Vaccination was associated with lower odds of RSV-related hospitalization (odds ratio [OR]: 0.23; 95% confidence interval [CI], 0.20–0.27; I² = 6.5%) and ED visits (OR: 0.23; 95% CI, 0.21–0.27; I² = 0.0%). Effectiveness against RSV-related hospitalization was lower in immunocompromised adults (OR: 0.31; 95% CI, 0.27–0.34; I² = 0.0%) than in immunocompetent individuals (OR: 0.20; 95% CI, 0.11–0.35; I² = 0.0%). Effectiveness did not differ by age group (60–74 vs ≥75 years) or vaccine product.

**Conclusions:**

Vaccination provided substantial protection against RSV-related healthcare utilization among older adults. Continued surveillance and real-world evidence are needed to inform immunization policy and improve protection in immunocompromised individuals.

## Teaser

In this meta-analysis of post-licensure observational studies involving around 1.5 million older adults, RSV vaccination showed substantial protection against RSV-related hospitalization (77%) and ED visits (77%). Effectiveness against RSV-related hospitalization was lower in immunocompromised (69%) versus immunocompetent individuals (80%).

Respiratory syncytial virus (RSV) is a leading cause of lower respiratory tract infections (LRTI) in older adults worldwide [1], contributing to substantial morbidity and healthcare costs [2–4]. In 2019 alone, an estimated 5.2 million RSV-related acute respiratory infections occurred among older adults aged 60 years or older in high-income countries, leading to approximately 470 000 hospitalizations and 33 000 in-hospital deaths [5]. In low- and middle-income countries, reported incidence among adults aged ≥65 years ranges from 10 to 178 episodes per 1000 person-years, with case-fatality rates of up to 27% among those older than 60 years [6]. A substantial portion of this burden is borne by individuals who are immunocompromised or experiencing comorbidities [7, 8]. Given the global burden of RSV and the vulnerability of high-risk populations, vaccination represents an important strategy for reducing RSV-related morbidity and mortality. Effective RSV prevention may also reduce antibiotic use for respiratory illness, thereby helping to mitigate antimicrobial resistance [9].

Recently, 3 RSV vaccines including RSVpreF (an unadjuvanted bivalent prefusion F protein-based vaccine), RSVpreF3 (an adjuvanted recombinant prefusion F protein-based vaccine), and mRESVIA (an mRNA-based vaccine) have been licensed for individuals aged ≥60 years following demonstration of high efficacy against RSV-related LRTI in clinical trials [10–12]. However, the pivotal trials had few hospitalization or emergency department (ED) events, which precluded reliable estimation of vaccine efficacy against these outcomes. Following vaccine approval, RSV immunization programs were introduced in several countries, including the United States (US), Canada, the European Union, and the United Kingdom (UK). Early observational studies have reported substantial reductions in RSV-related hospitalizations in populations where immunization has been implemented [13, 14]. However, real-world vaccine effectiveness may vary by age, vaccine type, and underlying health status.

We conducted a meta-analysis of post-licensure RSV immunization programs to quantify the real-world effectiveness (RWE) of prefusion F protein-based vaccines (RSVpreF and RSVpreF3). Although 2 systematic reviews have synthesized RWE of vaccines [15, 16], they relied on limited data from 3 studies published before April 2025 and did not evaluate high-risk subpopulations or differences in vaccine products. An updated and more comprehensive synthesis is therefore needed to inform evidence-based RSV immunization policy for older adults.

## METHODS

This study followed the updated Preferred Reporting Items for Systematic Reviews and Meta-Analyses (PRISMA) guidelines and the Meta-analyses of Observational Studies in Epidemiology (MOOSE) checklist [17, 18]. The protocol was prospectively registered in PROSPERO (CRD420251021777).

### Search Strategy and Selection Criteria

We conducted a comprehensive literature search of MEDLINE (Ovid), Embase (Ovid), Web of Science, Scopus, Global Health, and *medRxiv* preprint repository from 1 January 2023 to 30 December 2025 to identify studies reporting RWE of RSVpreF (Abrysvo) and RSVpreF3 (Arexvy), with no restrictions on language or study design. For this review, RWE studies were defined as observational investigations assessing RSV vaccine effectiveness in routine clinical practice across diverse settings, in contrast to the controlled conditions of clinical trials. Accordingly, mRESVIA was excluded because no published real-world observational studies evaluating its effectiveness were available when conducting this review. The search strategy combined Medical Subject Headings (MeSH) and free-text terms related to RSV and RSV vaccines (Supplementary Appendix pp 2–5). The search strategy was refined iteratively and finalized in accordance with the Peer-Review of Electronic Search Strategies (PRESS) guidelines [19]. Reference lists of included studies were also screened.

All retrieved studies were managed and deduplicated using EndNote 20, and imported into Covidence for screening. Two authors (D. M. S. and C. S.) independently assessed titles, abstracts, and full texts, with discrepancies resolved by consulting a third reviewer (S. M. M.). We included observational studies with prospective or retrospective cohort or case-control designs, reporting RWE of RSVpreF and RSVpreF3 among adults aged ≥60 years. Prespecified outcomes included RSV-related hospitalizations, ED visits, intensive care unit (ICU) admission, length of hospital stay (LoHS), incidence of RSV-related LRTI, and mortality. Meta-analysis was conducted only for outcomes that were sufficiently and consistently reported; other outcomes with limited data or heterogeneity in definitions were synthesized narratively. Conference abstracts were excluded owing to insufficient information for quality assessment. For overlapping populations or datasets, only the most recent and larger study was retained. Studies focused exclusively on safety and adverse events, or employing ineligible designs (eg, clinical trials/reviews, economic evaluations, and modeling analyses) were excluded. Definitions of primary outcomes, key terminology, the rationale for subgroup analyses, and details of inclusion and exclusion criteria are provided in the Supplementary Appendix (pp 5–8, Table 1).

### Data Extraction and Quality Assessment

Two reviewers (D. M. S. and C. S.) independently extracted data using a framework adapted from our recent study on the effectiveness of nirsevimab against RSV-related outcomes in infants [20]. Key study characteristics, including first author, year of publication, study design, country, setting, and population demographics, were recorded into Microsoft Excel 365. In this study, “vaccination” is defined as an exposure based on vaccination status (receipt vs non-receipt) assessed prior to outcome occurrence and does not represent a cumulative vaccination history. For dichotomous outcomes, data were entered into a 2 × 2 contingency table, with the numerator representing the number of events and the denominator representing the total number of participants in vaccinated and control groups. When vaccine effectiveness was reported as 1 − OR × 100%, the corresponding odds ratio (OR) and 95% confidence intervals (CIs) were derived using the formula 1 − (effectiveness/100). When both crude and adjusted estimates were available, adjusted values were extracted. The covariates included in each study's adjusted model are summarized in the Supplementary Appendix (Table 2). Because event rates were <10% in all vaccinated arms, hazard ratios, risk ratios, and ORs were treated as numerically comparable and included in pooled analyses [21].

The same reviewers independently assessed study quality using the Joanna Briggs Institute (JBI) Critical Appraisal Checklist for Observational Studies [22]. The JBI checklist comprises 10 domains for case-control studies and 11 domains for cohort studies, with responses categorized as yes, no, unclear, or not applicable. As no formal scoring system is prescribed in the JBI checklist, studies were classified as having a low risk of bias if >75% of responses were yes, moderate risk with 50%–75%, and high risk with <50%. The certainty of evidence was assessed following the Grading of Recommendations, Assessment, Development and Evaluations (GRADE) framework [23].

### Data Analyses

Statistical analyses were performed using the “*metan*” package in Stata/SE (version 16.1). For dichotomous outcomes such as RSV-related hospitalization and ED visits, pooled ORs were calculated to estimate vaccine effectiveness. Reported ORs and 95% CIs were log-transformed, with standard errors (SEs) derived as (upper limit − lower limit)/3.92 [24]. Pooled estimates were back-transformed for interpretability and presented in forest plots. Where meta-analysis was not feasible because of limited data or heterogeneity in outcome definitions (eg, ICU admission, incidence of RSV-related LRTI, mortality), results were synthesized narratively (Supplementary Appendix, Table 9). Mortality and LoHS could not be assessed because these outcomes were not reported in the included studies.

To address between-study variability, we performed inverse-variance random-effects meta-analyses using the Mandel–Paule *τ*² estimator [25]. The Hartung-Knapp-Sidik-Jonkman adjustment was applied to account for uncertainty arising from study sample sizes and event-rate variability [26]. Heterogeneity was assessed using the I² and *τ*^2^ metrics, interpreted according to Cochrane guidance (I² ≤ 40% low heterogeneity, 41%–75% moderate heterogeneity, and >75% high heterogeneity). Additionally, we report 95% prediction intervals (PIs) for key outcomes, which estimate the range in which vaccine effectiveness from a future study is likely to fall.

Subgroup analyses were conducted when at least 3 studies were available to assess heterogeneity by immune status and age. Vaccine type emerged as an important variable and was examined in a post hoc exploratory subgroup analysis. Meta-regression was conducted in R using the “*meta*” package to examine whether study timeframe or case definition stringency (laboratory-confirmed RSV only vs clinical syndrome with laboratory-confirmed RSV) moderated the effect size for RSV-related hospitalizations. Publication bias was evaluated through funnel plot symmetry and the Egger regression test when 4 or more studies were available, with a *P*-value <.05 indicating bias. Sensitivity analyses tested the robustness of the findings by (1) excluding cohort studies, (2) replacing the Mandel–Paule estimator with the DerSimonian and Laird method, (3) removing studies with large SEs, and (4) replacing a 2-season (2023–2024 and 2024–2025) study with its earlier single-season (2023–2024) version.

## RESULTS

### Identification and Selection of Studies

**Figure 1.**
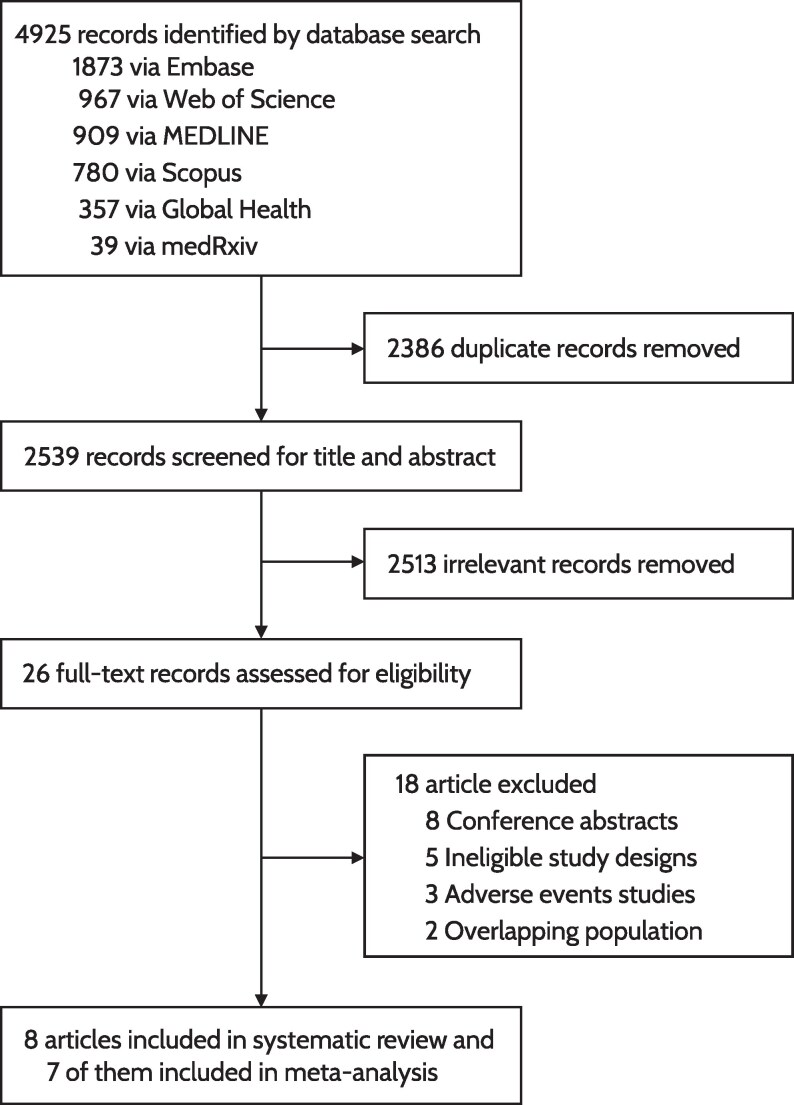
PRISMA flowchart of study selection process.

We identified 4925 potentially eligible records, of which 2539 unique citations underwent title and abstract screening after removing duplicates (Figure 1). Of these, 26 articles (1.02%) were retrieved for full-text screening. Sixteen studies were subsequently excluded because they were conference abstracts (n = 8), used ineligible study designs (n = 5), or reported only adverse events (n = 3) (Supplementary Appendix, Table 3). Two additional studies were excluded due to overlapping populations with the more recent, larger participant analysis. In total, 8 studies met the inclusion criteria, representing approximately 1.5 million participants.

### Characteristics of the Studies

The included studies were conducted in 2 countries: 7 in the US [14, 27–32] and 1 in the UK [33]. Of the 7 US studies, 6 were conducted during the 2023–2024 RSV season, and 1 spanned across both the 2023–2024 and 2024–2025 seasons [31]. The UK study was undertaken during the 2024–2025 season as the first RSV season with vaccination following authorization. Six studies evaluated both RSVpreF and RSVpreF3 [14, 28–31, 34], and 2 assessed only RSVpreF [32, 33]. Seven studies were multicenter, using electronic health records from multiple sites, and 1 study was single-center. Participants were predominantly female. Respiratory syncytial virus diagnosis was primarily based on reverse transcription-polymerase chain reaction. The dataset comprised 2 cohort [29, 34] and 6 case-control studies [14, 28, 30–33], all assessed as low risk of bias (Supplementary Appendix, Tables 4 and 5). Study characteristics are summarized in Table 1.

**Table 1.** Study Characteristics

Study	Country	Study Design	Sample Size	Sex (M/F)%	Median Age, Year	Timeframe	Testing Method	Vaccine Type	Outcomes Reported
Bajema et al **[27]**	United States	Retrospective cohort	576 222	94/6	76	September 2023 to March 2024	RT-PCR	RSVpreF and RSVpreF3	RSV-H, RSV-EDV, ICU-A, and RSV-I
Fry et al **[28]**	United States	Test-negative case-control	787 822	NR	74	October 2023 to April 2024	RT-PCR or RAT	RSVpreF and RSVpreF3	RSV-H, RSV-EDV, and RSV-MA-RI
Payne et al **[30]**	United States	Test-negative case-control	37 842	47/53	76	October 2023 to March 2024	RT-PCR or RAT	RSVpreF and RSVpreF3	RSV-H, RSV-EDV
Tartof et al **[32]**	United States	Test-negative case-control	8965	45/55	77	November 2023 to April 2024	RT-PCR	RSVpreF	RSV-H, RSV-EDV
Surie et al **[14]**	United States	Test-negative case-control	2978	49/51	72	October 2023 to March 2024	RT-PCR	RSVpreF and RSVpreF3	RSV-H
Surie et al **[31]**	United States	Test-negative case-control	6958	48/52	72	October 2023 to March 2024, October 2024 to April 2025	RT-PCR	RSVpreF and RSVpreF3	RSV-H
Symes et al **[33]**	United Kingdom	Test-negative case-control	1006	47/53	77	October 2024 to March 2025	RT-PCR	RSVpreF	RSV-H, OxU
Godonou et al **[29]**	United States	Prospective cohort	281	29/71	67	August 2023 to March 2024	RT-PCR	RSVpreF and RSVpreF3	RSV-I

RT-PCR, reverse transcription polymerase chain reaction; RAT, rapid antigen tests; RSV-H, respiratory syncytial virus-related hospitalization; RSV-EDV, respiratory syncytial virus-related emergency department visits; ICU-A, intensive care unit admission; RSV-I, respiratory syncytial virus infection incidence; RSV-MA-RI, respiratory syncytial virus-related medically attended respiratory illness; OxU, oxygen use; NR, not reported; M, male; F, female.

### Respiratory Syncytial Virus–Related Hospitalization and Emergency Department Visits

**Figure 2.**
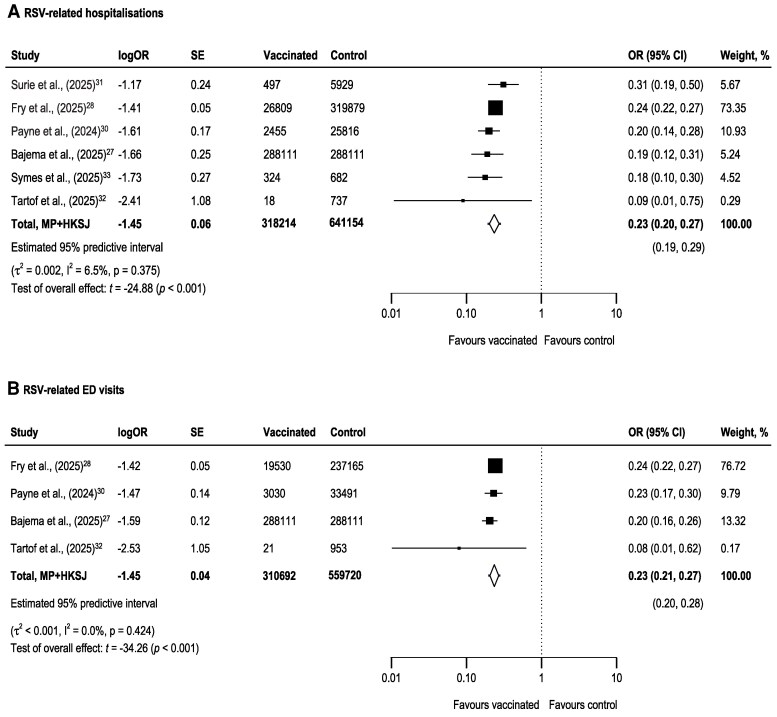
Pooled estimates of the association between vaccination and (*A*) RSV-related hospitalization and (*B*) emergency department (ED) visits among older adults. Forest plots show pooled odds ratios (OR) with 95% confidence intervals (CI). Heterogeneity is presented as τ² (between-study variance) and I² (percentage of total variability attributable to heterogeneity). SE, standard error; MP, Mandel–Paule; HKSJ, Hartung-Knapp-Sidik-Jonkman adjustment.

Of the 7 studies reporting RSV-related hospitalization, 6 were eligible for inclusion in the primary analysis. In total, the included studies comprised 318 214 vaccinated adults aged ≥60 years and 641 154 controls (Figure 2*A*). The pooled analysis revealed that vaccination was associated with significantly lower odds of RSV-related hospitalization compared to controls (OR: 0.23, 95% CI: 0.20–0.27; *P* < mpa#thinsp;.001; 95% PI: 0.19–0.29), with low between-study heterogeneity (I^2^ = 6.5%; *τ*^2^ = 0.002).

Four studies reported RSV-related ED visits, with 310 692 vaccinated and 559 720 controls (Figure 2*B*). Vaccination was associated with significantly lower odds of RSV-related ED visits (OR: 0.23; 95% CI, 0.21–0.27; *P* < .001; 95% PI: 0.20–0.28), with low between-study heterogeneity (I² = 0.0%; *τ*² < 0.001). The overall certainty of evidence for RSV-related hospitalizations and ED visits, corresponding to the GRADE evaluation of included studies, is summarized in the Supplementary Appendix (Table 8).

### Subgroup Analysis

**Figure 3.**
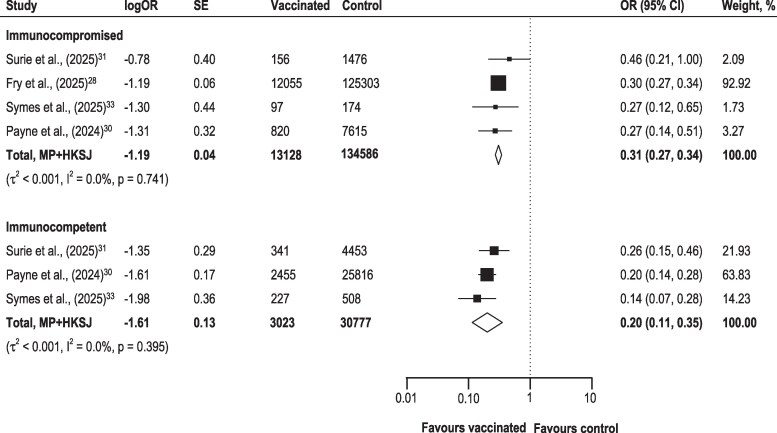
Pooled estimates of the association between vaccination and RSV-related hospitalization by immune status. Forest plots show pooled odds ratios (OR) with 95% confidence intervals (CI). Heterogeneity is presented as τ² (between-study variance) and I² (percentage of total variability attributable to heterogeneity). SE, standard error; MP, Mandel–Paule; HKSJ, Hartung-Knapp-Sidik-Jonkman adjustment.

Among studies reporting immune status, 81.4% of participants were immunocompromised (13 128 vaccinated and 134 586 unvaccinated), and 18.6% were immunocompetent (3023 vaccinated and 30 777 unvaccinated). When comparing vaccinated and control groups by immune status (Figure 3), the Kruskal–Wallis test indicated a significant difference in pooled estimates between subgroups (*P* = mpa#thinsp;.034). Vaccination was associated with lower odds of hospitalization among immunocompetent individuals (OR: 0.20, 95% CI, 0.11–0.35) than immunocompromised individuals (OR: 0.31, 95% CI, 0.27–0.34). Heterogeneity was low in both subgroups (I^2^ = 0.0%; *τ*^2^ < 0.001).

When comparing older adults aged 60–74 years with those aged ≥75 years (Supplementary Appendix, Figure 1), we found no significant differences (Kruskal–Wallis test, *P* = mpa#thinsp;.376) in RSV-related hospitalization. Among those aged 60–74 years, the pooled OR for RSV-related hospitalization was 0.27 (95% CI, 0.10–0.71), with moderate heterogeneity (I² = 55.9%, *τ*² = 0.099). Among older adults aged ≥75 years, the pooled OR was 0.24 (95% CI, 0.20–0.27), with low heterogeneity (I² = 0.0%, *τ*² < 0.001).

Subgroup analyses by vaccine type (Supplementary Appendix, Figure 2) showed no significant differences in effectiveness for preventing RSV-related hospitalization between RSVpreF and RSVpreF3 (Kruskal–Wallis, *P* = mpa#thinsp;.643). The pooled OR was 0.17 (95% CI, 0.10–0.31) for RSVpreF3 and 0.23 (95% CI, 0.13–0.41) for RSVpreF. Heterogeneity was low for both RSVpreF (I² = 2.7%, *τ*² = 0.003) and RSVpreF3 (I² = 0.0%, *τ*² < 0.001) vaccines.

### Sensitivity Analysis

Sensitivity analyses demonstrated that the results of primary analysis were robust (Supplementary Appendix, Tables 6 and 7). Vaccination remained associated with lower odds of RSV-related hospitalization when cohort studies were removed (OR: 0.24; 95% CI, 0.19–0.29), when studies with high SEs were excluded (OR: 0.23; 95% CI, 0.19–0.28), and when the DerSimonian and Laird method was used in place of the Mandel–Paule estimator (OR: 0.23; 95% CI, 0.20–0.27). We also performed a sensitivity analysis in which the updated study spanning 2 RSV seasons (2023–2024 and 2024–2025) was replaced with its earlier version limited to the 2023–2024 season. This substitution yielded an OR of 0.24 (95% CI, 0.21–0.26) for RSV-related hospitalization among vaccinated individuals, consistent with the primary analysis. Heterogeneity was low across these analyses.

Vaccination was associated with lower odds of RSV-related ED visits when cohort studies were removed (OR: 0.24; 95% CI, 0.20–0.28), when outlier studies were excluded (OR: 0.24; 95% CI, 0.20–0.28), and when the DerSimonian–Laird method replaced the Mandel–Paule estimator (OR: 0.23; 95% CI, 0.21–0.27), with low heterogeneity observed in each scenario (Supplementary Appendix, Table 6). In addition, sensitivity analyses by subgroups supported the robustness of the primary findings for RSV-related hospitalization. After excluding outlier studies, pooled estimates for immunocompromised participants, the 60–74-year age group, and vaccine type remained consistent with the primary analyses, with low heterogeneity observed across all scenarios (Supplementary Appendix, Table 7).

### Meta-regression and Publication Bias

No significant association was observed between study duration and the estimated effect size (*P* = mpa#thinsp;.125), indicating that differences in study duration did not materially affect the pooled estimates (Supplementary Appendix, Figure 3). Meta-regression using case-definition stringency as a study-level covariate also showed no association with effect estimates for RSV-related hospitalizations (*P* = mpa#thinsp;.374), indicating that variation in case-definition stringency did not influence the pooled estimates (Supplementary Appendix, Figure 4).

Publication bias was assessed using funnel plots and the Egger test for both RSV-related hospitalization (*P* = mpa#thinsp;.156) and RSV-related ED visits (*P* = mpa#thinsp;.060), with no evidence of bias (Supplementary Appendix, Figures 5 and 6).

## DISCUSSION

This study synthesizes real-world evidence on the effectiveness of RSV vaccination in older adults, drawing on 8 post-licensure observational studies involving nearly 1.5 million participants. Our findings demonstrate that vaccination was associated with substantial protection, with pooled effectiveness of 77% against RSV-related hospitalization and 77% against RSV-related ED visits. Effectiveness in preventing RSV-related hospitalization was consistent across age groups and vaccine products, but lower among immunocompromised individuals (69%) than immunocompetent adults (80%).

Reduced effectiveness in immunocompromised individuals likely reflects impaired humoral and cellular immune responses. Several studies show that approximately 40% of immunocompromised adults fail to seroconvert or reach conservative neutralization thresholds after RSV vaccination [35–37], whereas immunocompetent adults typically achieve near-universal seroconversion and a 10-fold rise in pre-F IgG titers [10, 35, 38, 39]. Immunosuppressive therapies further attenuate responses; for example, lung transplant recipients receiving mycophenolate mofetil show markedly reduced RSV-specific antibody and CD4 T-cell responses following vaccination [36, 37, 40]. However, effectiveness estimates in this subgroup should be interpreted cautiously, as definitions of immunocompromised status varied across studies and may encompass biologically diverse conditions. This heterogeneity, together with underlying immune dysregulation and immunosuppressive therapies, likely contributes to the reduced vaccine effectiveness observed in this subgroup compared with the immunocompetent individuals.

A previous meta-analysis, based on 3 early observational studies, reported RSV vaccine effectiveness at 79.6% against hospitalization and 77.9% against ED visits [15]. Another meta-analysis of the same early studies reported 79% effectiveness against RSV-related hospitalization [16]. While broadly consistent with those early results, our study incorporates additional post-licensure data from substantially larger populations and evaluates key subgroups by age, immune status, and vaccine product, providing a more comprehensive and current assessment of RSV vaccine effectiveness in older adults.

## LIMITATIONS

This study has several limitations. First, all included post-licensure observational studies were conducted in the United States and the United Kingdom, which may limit the generalizability to settings with different healthcare access and care-seeking behavior, population risk profiles, testing practices, or vaccine delivery systems. Second, vaccination status was ascertained using immunization registries, electronic health records, or medical claims, which may incompletely capture RSV vaccine receipt and introduce exposure misclassification [41]. Third, studies varied in design, population characteristics, case definitions, and analytic methods. Most studies defined RSV-related hospitalizations and ED visits using clinical syndromes with laboratory confirmation, whereas 1 study relied on laboratory confirmation alone. Although broader syndrome-based definitions may dilute effect estimates, our subgroup, sensitivity, and meta-regression analyses yielded consistent results, supporting the robustness of the primary findings. Importantly, for clinical decision-making, the relatively narrow 95% PIs further suggest that effectiveness estimates are likely to be comparable across healthcare settings and surveillance definitions within the populations studied. Fourth, limited data for some RSV outcomes prevented pooled analysis, and these outcomes were synthesized narratively. Similarly, insufficient data precluded subgroup analyses by major comorbidities. Furthermore, vaccine safety was outside the scope of this analysis and too few studies reporting safety outcomes to permit pooled quantitative assessment. Fifth, although differences in participant enrollment periods and follow-up durations could theoretically influence vaccine effectiveness, meta-regression found no evidence that study timeframe materially affected these estimates. Sixth, definitions of immunocompromised status varied across studies, relying on ICD-coded diagnoses or clinical criteria, which may have contributed to heterogeneity and influenced pooled estimates. In addition, the relatively small number of immunocompetent participants limited the precision of subgroup-specific estimates. Finally, most cohorts comprised a higher proportion of female participants. Although sex-adjusted estimates were extracted when available, generalizability of results to predominantly male populations may be limited.

## CONCLUSION

Advances in RSV prevention over the past 2 decades have led to the introduction of long-acting monoclonal antibodies [20, 42] and vaccines to protect infants and older adults from RSV disease. In this context, our study provides real-world evidence that the benefits of RSVpreF and RSVpreF3 observed in clinical trials effectively translate into reduced healthcare utilization. Overall, our results indicate that RSV vaccination offers substantial protection against serious RSV-related outcomes in older adults, supporting its integration into geriatric immunization programs to reduce disease burden and alleviate pressures on healthcare systems during seasonal epidemics.

## Supplementary Material

ciag107_Supplementary_Data

## Supplementary Data

Supplementary materials are available at *Clinical Infectious Diseases* online. Consisting of data provided by the authors to benefit the reader, the posted materials are not copyedited and are the sole responsibility of the authors, so questions or comments should be addressed to the corresponding author.
