# Supplementary material for: Real-World Effectiveness of RSVpreF and RSVpreF3 Vaccines in Preventing Hospitalization and Emergency Department Visits Associated With Respiratory Syncytial Virus in Older Adults: A Meta-analysis
Source: Clin Infect Dis. 2026 Feb 18;82(5):e1104–11. doi: 10.1093/cid/ciag107 (PMC13189658; doi:10.1093/cid/ciag107)
Supplement: ciag107_Supplementary_Data [file ciag107_supplementary_data.docx]

**Appendix A: Supplementary Data**

**Real-World Effectiveness of RSVpreF and RSVpreF3 Vaccines in Preventing Hospitalisation and Emergency Department Visits Associated with Respiratory Syncytial Virus in Older Adults: A Meta-Analysis**

**Dewan Md. Sumsuzzman,^1^ Congjie Shi,^2,3^ Seyed M. Moghadas^1^**

^1^ Agent-Based Modelling Laboratory, Centre of Excellence in AI for Public Health Advancement, York University, Toronto, ON M3J 1P3, Canada

^2^ Laboratory for Industrial and Applied Mathematics, York University, Toronto, ON M3J 1P3, Canada

^3^ National Laboratory for Health Security, University of Szeged, 6720 Szeged, Hungary

**TABLE OF CONTENTS**

**SEARCH STRATEGY 2**

**SUPPLEMENTARY METHODS 5**

Eligibility criteria 5

Rationale and hypotheses for subgroup analyses 6

**SUPPLEMENTARY TABLES 6**

Table S1. Study-level definitions of respiratory syncytial virus-related hospitalisation, emergency department visits, and immunocompromised populations. 6

Table S2. Adjusted covariates included in multivariable models across studies in the meta‑analysis. 9

Table S3. Excluded studies in full-text review. 10

Table S4: Quality assessment of included cohort studies using the JBI critical appraisal tool. 12

Table S5: Quality assessment of included case-control studies using the JBI critical appraisal tool. 13

Table S6. Sensitivity analyses for main outcomes. 14

Table S7. Sensitivity analysis of specific subgroups for RSV-related hospitalisations. 15

Table S8. The overall certainty of RSV-related hospitalisations and ED visits using GRADE.. 16

Table S9. Narrative synthesis of vaccine effectiveness estimates against incidence of RSV‑related LRTI and ICU admission from included studies. 17

**SUPPLEMENTARY FIGURES 18**

Figure S1. Subgroup analysis of RSV-related hospitalisation by age, comparing RSV-vaccinated individuals with controls. 18

Figure S2. Subgroup analysis of RSV-related hospitalisation by vaccine type, comparing RSV-vaccinated individuals with controls. 19

Figure S3. Bubble plot from meta-regression examining the association between study duration (in days) and RSV-related hospitalisations. 20

Figure S4. Bubble plot from meta-regression examining the association between case definition stringency and RSV-related hospitalisations. 21

Figure S5. Funnel plot of studies reporting RSV-related hospitalisations for older adults aged 60 years or older. 22

Figure S6. Funnel plot of studies reporting RSV-related emergency department visits for older adults aged 60 years or older. 23

**References 24**

# **SEARCH STRATEGY**

**Database:** Ovid MEDLINE(R) and Epub Ahead of Print, In-Process, In-Data-Review & Other Non-Indexed Citations, <*January 01, 2023 to December 30, 2025*>

1 exp Respiratory Syncytial Viruses/ 12040

2 (respiratory syncytial virus or RSV).ti,ab,kf. 25915

3 1 or 2 27110

4 exp Respiratory Tract Infections/ 702567

5 ('Respiratory Tract Infections' or 'Respiratory Infections').ti,ab. 38932

6 4 or 5 720680

7 exp Pneumonia/ 408631

8 exp Bronchiolitis/ 10558

9 3 or 6 or 7 or 8 741023

10 exp Respiratory Syncytial Virus Vaccines/ 1348

11 (RSVpreF or RSVpreF3 or Prefusion F or pre-F protein or Prefusion Protein F3 or pre-fusion F or abrysvo or arexvy or mResvia or rsv vaccine or rsv vaccines or respiratory syncytial virus vaccine or respiratory syncytial virus vaccines).ti,ab. 1633

12 10 or 11 2199

13 9 and 12 2146

14 limit 13 to yr="2023 -Current" 909

**Database:** Embase <*January 01, 2023 to December 30, 2025*>

1 exp Respiratory Syncytial Viruses/ 26745

2 (respiratory syncytial virus or RSV).ti,ab,kf. 34278

3 1 or 2 41163

4 exp Respiratory Tract Infections/ 571306

5 ('Respiratory Tract Infections' or 'Respiratory Infections').ti,ab. 56898

6 4 or 5 583038

7 exp Pneumonia/ 458296

8 exp Bronchiolitis/ 30375

9 3 or 6 or 7 or 8 885296

10 exp Respiratory Syncytial Virus Vaccines/ 3640

11 exp "pf 06928316"/ 226

12 exp gsk 3844766a/ 111

13 (abrysvo or pf 6928316 or pf06928316 or pf6928316).ti,ab. 101

14 (arexvy or gsk3844766a).ti,ab. 76

15 (RSVpreF or RSVpreF3 or Prefusion F or pre-F protein or Prefusion Protein F3 or pre-fusion F or mResvia or rsv vaccine or rsv vaccines or respiratory syncytial virus vaccine or respiratory syncytial virus vaccines).ti,ab. 2148

16 10 or 11 or 12 or 13 or 14 or 15 4259

17 9 and 16 4051

18 limit 17 to yr="2023 -Current" 1873

**Database:** Global Health <*January 01, 2023 to December 30, 2025*>

1 respiratory syncytial virus.ti,ab. 11076

2 RSV.ti,ab. 9151

3 1 or 2 13045

4 Respiratory Tract Infections.ti,ab. 10935

5 Respiratory Infections.ti,ab. 12312

6 4 or 5 21878

7 exp Pneumonia/ 52034

8 exp Bronchiolitis/ 2704

9 3 or 6 or 7 or 8 81130

10 (Respiratory Syncytial Virus Vaccines or RSV Vaccines or RSV vaccination).ti,ab. 373

11 (RSVpreF or RSVpreF3 or Prefusion F or pre-F protein or Prefusion Protein F3 or pre-fusion F or abrysvo or arexvy or mResvia or rsv vaccine or rsv vaccines or respiratory syncytial virus vaccine or respiratory syncytial virus vaccines).ti,ab. 679

12 10 or 11 749

13 9 and 12 742

14 limit 13 to yr="2023 -Current" 357

**Database:** *Web of Science Core Collection* <*January 01, 2023 to December 30, 2025*>

| **#** | **Search Query** | **Results** |
| --- | --- | --- |
| 1 | (ALL=(Respiratory Syncytial Viruses)) OR TS=((Respiratory Syncytial Viruses OR RSV)) | 35257 |
| 2 | ((ALL=(Respiratory Tract Infections)) OR TI=((Respiratory Tract Infections OR Respiratory Infections))) OR AB=((Respiratory Tract Infections OR Respiratory Infections)) | 165508 |
| 3 | ALL=(Pneumonia) | 208690 |
| 4 | ALL=(Bronchiolitis) | 20310 |
| 5 | #1 OR #2 OR #3 OR #4 | 372965 |
| 6 | TS=(Respiratory Syncytial Virus Vaccines) | 5836 |
| 7 | (TI=((RSVpreF or RSVpreF3 or Prefusion F or pre-F protein or Prefusion Protein F3 or pre-fusion F or abrysvo or arexvy or mResvia or rsv vaccine or rsv vaccines or respiratory syncytial virus vaccine or respiratory syncytial virus vaccines))) OR AB=((RSVpreF or RSVpreF3 or Prefusion F or pre-F protein or Prefusion Protein F3 or pre-fusion F or abrysvo or arexvy or mResvia or rsv vaccine or rsv vaccines or respiratory syncytial virus vaccine or respiratory syncytial virus vaccines)) | 4438 |
| 8 | #6 OR #7 | 6277 |
| 9 | #5 AND #8 | 6166 |
| 10 | TS=("population groups" NOT "animal models") OR (TS=(men OR women OR patient OR female OR male OR subjects OR adult) NOT TS="animal models") | 16277477 |
| 11 | #9 AND #10 | 2207 |
| 12 | #9 AND #10 Timespan: 2023-01-01 to 2025-10-21 | 967 |

**Database:** *Scopus <January 01, 2023 to December 30, 2025>*

( INDEXTERMS ( "respiratory syncytial viruses" ) OR TITLE-ABS ( "respiratory syncytial viruses" ) OR TITLE-ABS ( "RSV" ) OR INDEXTERMS ( "respiratory tract infections" ) OR TITLE-ABS ( "respiratory tract infections" ) OR TITLE-ABS ( "respiratory infections" ) OR INDEXTERMS ( "pneumonia" ) OR INDEXTERMS ( "bronchiolitis" ) ) AND ( TITLE-ABS ( "RSVpreF" ) OR TITLE-ABS ( "RSVpreF3" ) OR TITLE-ABS ( "Prefusion F" ) OR TITLE-ABS ( "pre-F protein" ) OR TITLE-ABS ( "Prefusion Protein F3" ) OR TITLE-ABS ( "pre-fusion F" ) OR TITLE-ABS ( "Abrysvo" ) OR TITLE-ABS ( "Arexvy" ) OR TITLE-ABS ( "mResvia" ) OR TITLE-ABS ( "RSV vaccine" ) OR TITLE-ABS ( "RSV vaccines" ) OR TITLE-ABS ( "respiratory syncytial virus vaccine" ) OR TITLE-ABS ( "respiratory syncytial virus vaccines" ) ) AND PUBYEAR > 2023 AND PUBYEAR < 2025 =780

# **SUPPLEMENTARY METHODS**

### **Eligibility criteria**

1. **Inclusion criteria:**

- *Population*: Individuals aged 60 years or older, regardless of sex or study settings (e.g., outpatient, inpatient, emergency, and long-term care facility), who are at risk of severe acute respiratory infection caused by respiratory syncytial virus (RSV).
- *Intervention:* Studies evaluating the real-world effectiveness of any licensed RSV vaccine in preventing RSV-related outcomes, such as hospitalisation, intensive care unit admission, and emergency department visits.
- *Comparison:* We included studies that compared vaccine effectiveness between RSV-vaccinated versus unvaccinated (control) individuals.
- *Outcomes:* Studies reporting on at least one the following outcomes: RSV-related hospitalisation, RSV-related emergency department visits.
- *Study design*: We included longitudinal cohort studies, studies using routinely collected healthcare data, test–negative case–control studies, and matched case–control cohort studies in our meta-analysis.

1. **Exclusion criteria:**

- Studies evaluating the immunogenicity or underlying mechanisms of RSV vaccine response.
- Studies assessing vaccine uptake and caregiver perceptions, knowledge, and attitudes regarding RSV vaccination.
- Studies evaluating the co-administration of RSV vaccines with COVID-19 or influenza vaccines.
- Studies that reported solely adverse events from post-marketing surveillance studies.
- Ineligible study designs (such as mathematical modelling, simulation studies, case reports, case series, editorials, letter to editor, reviews, conference abstracts, clinical trials, and population-level regression discontinuity analyses).
- When overlapping populations or datasets were identified, only the most recent studies with the largest sample sizes were included; earlier versions of studies were excluded to prevent duplication.

### **Rationale and hypotheses for subgroup analyses**

- **Age:** Examined because immunosenescence and age-related changes in RSV risk and vaccine/immune response in older adults. We hypothesised that vaccine effectiveness could vary across older age strata.
- **Immune status:** Immunocompromised status was examined as a prespecified effect‑modifying factor, given established evidence of impaired immune responses to vaccination in this population [1]; we therefore hypothesised lower vaccine effectiveness compared with immunocompetent individuals.
- **Vaccine type:** Examined to explore whether differences in vaccine formulation or platform might contribute to between-study heterogeneity in effectiveness estimates. This analysis was conducted as a post-hoc exploratory assessment.

# **SUPPLEMENTARY TABLES**

### **Table S1.** Study-level definitions of respiratory syncytial virus-related hospitalisation, emergency department visits, and immunocompromised populations.

| Study | Definition summary | Case definition approach |
| --- | --- | --- |
| RSV-related hospitalisations | | |
| Bajema et al.,(2025) [2] | RSV-related hospitalisations were defined as inpatient admissions that occurred within one day before or after a positive RSV test result. | Lab-confirmed only |
| Fry et al., (2025) [3] | RSV-related hospitalisation was defined as a hospital admission for ARI in which the patient had a positive RSV test result from clinical viral testing (including RT-PCR, nucleic acid amplification, or antigen testing) that was performed within 10 days before or through 3 days after the ARI-related hospitalisation. | Clinical syndrome + Lab-confirmed RSV |
| Payne et al., (2024) [4] | RSV-related hospitalisation was defined as an inpatient admission lasting more than 24 hours with laboratory-confirmed RSV infection and a diagnosis consistent with RSV-like illness. A positive RSV test (molecular or viral culture; positive antigen tests were included; negative antigen tests were excluded due to low sensitivity) had to be documented within 10 days before or through less than 72 hours after the hospitalisation. | Clinical syndrome + Lab-confirmed RSV |
| Surie et al., (2025) [5] | RSV-related hospitalisation was defined as hospital admission for ARI in which the patient had a laboratory-confirmed RSV infection. RSV infection could be detected either by clinical viral testing conducted at the enrolling hospital or by central laboratory testing (RT-PCR) of nasal swabs collected at enrollment. Eligible RSV tests were those performed within 10 days of illness onset and within 3 days of hospital admission. | Clinical syndrome + Lab-confirmed RSV |
| Symes et al.,(2025) [6] | RSV-related hospitalisation was defined as an inpatient admission lasting ≥24 hours for ARI, with laboratory-confirmed RSV infection by molecular testing (e.g., RT-PCR) of nasopharyngeal or combined nose and throat swabs collected within 48 hours of hospital admission. | Clinical syndrome + Lab-confirmed RSV |
| Tartof et al., (2025) [7] | RSV-related hospitalisation was defined as an ARI hospitalisation in which a nasal or nasopharyngeal specimen tested positive for RSV and was collected from 14 days before through 3 days after hospital admission. | Clinical syndrome + Lab-confirmed RSV |
| RSV-related ED visits | | |
| Bajema et al., (2025) [2] | RSV-related ED or urgent care visits were defined as any encounter occurring within 1 day before or after the eligible positive RSV test result. | Lab-confirmed only |
| Fry et al., (2025) [3] | RSV-related ED or urgent care visits were defined as encounters for ARI, identified using ICD-10 codes (J00-J06, J09-J18, J20-J22, R05), with a positive RSV test performed within 10 days before or 3 days after the encounter. | Clinical syndrome (ICD-10) + Lab-confirmed RSV |
| Payne et al., (2024) [4] | RSV-related ED visits were defined as ED encounters with RSV-like illness among patients who underwent RSV testing. | Clinical syndrome + Lab-confirmed RSV |
| Tartof et al., (2025)[7] | RSV-related ED visits were defined as ED encounters with a positive RSV test from nasal or nasopharyngeal samples collected from 14 days before through 3 days after the encounter. | Clinical syndrome + Lab-confirmed RSV |
| Immunocompromised population | | |
| Fry et al., (2025) [3] | Immunocompromised status was determined from ICD-10-CM diagnostic codes indicating HIV infection, malignant neoplasms (solid or haematological), haematologic or immune deficiency disorders, inflammatory or rheumatologic conditions, chronic liver disease, or organ- and tissue-transplant–related codes. | ICD-10-CM–based definition |
| Payne et al., (2024) [4] | Individuals were considered immunocompromised if they had ICD-10 codes corresponding to any of six categories: (1) haematologic malignancy, (2) solid malignancy, (3) transplant recipient, (4) rheumatologic or inflammatory disorder, (5) congenital or acquired immune deficiency, or (6) HIV infection. | ICD-10–based definition |
| Surie et al., (2025) [5] | Defined moderate-to-severe immunocompromise as the presence of an active or recently treated malignancy (within 6 months), solid-organ or haematopoietic-cell transplant, HIV infection, primary immunodeficiency, recent use of immunosuppressive medication (past 30 days), or other medical conditions causing substantial immune suppression. | Clinically defined (treatment / condition based) |
| Symes et al., (2025) [6] | Participants were classified as immunocompromised if they were receiving chemotherapy or radiotherapy, had undergone solid-organ, bone-marrow, or stem-cell transplantation, had HIV infection or a genetic immune disorder, were on long-term immunosuppressive or biologic therapy, were receiving systemic corticosteroids ≥ 20 mg prednisolone equivalent for > 1 month, or required chronic immunosuppressive therapy for autoimmune or inflammatory disease. | Clinically defined (treatment/ diagnosis based) |

RSV, respiratory syncytial virus; ARI, acute respiratory infection; RT-PCR, reverse transcription–polymerase chain reaction; LRTD, lower respiratory tract disease; ED, emergency department; ICD-10, International Statistical Classification of Diseases and Related Health Problems-10th Revision; HIV, human immunodeficiency viruses.

### **Table S2.** Adjusted covariates included in multivariable models across studies in the meta‑analysis.

| **Study** | **Adjusted Covariates** |
| --- | --- |
| Bajema et al., (2025) [2] | Age, sex, race, ethnicity, underlying conditions, and geographic location |
| Fry et al., (2025) [3] | Age, month of RSV test, immunocompromised status, and state of residence |
| Payne et al., (2024) [4] | Age, race and ethnicity, sex, underlying medical conditions, Social Vulnerability Index, site, calendar  day, and geographical region |
| Tartof et al., (2025) [7] | Age, sex, encounter months, race/ethnicity, Charlson Comorbidity Index, and previous outpatient, inpatient encounters, and ED encounters |
| Surie et al., (2024) [8] | Age, sex, race and ethnicity, US Department of Health and Human Services region, and calendar month and year of admission. |
| Surie et al., (2025) [5] | Age, sex, race and ethnicity, US Department of Health and Human Services region, and calendar month and year of admission. |
| Symes et al., (2025) [6] | Age, sex, ethnicity, Index of Multiple Deprivation, site, and region |
| Godonou et al., (2025) [9] | Sex, race, ethnicity, and the presence of high-risk health conditions |

### **Table S3.** Excluded studies in full-text review.

| Sl/No. | References | Reasons for exclusion |
| --- | --- | --- |
| 1. | Li J, Zhang Z, Wang M. Post-licensure safety of respiratory syncytial virus vaccines, Vaccine Adverse Event Reporting System, United States, May 2023-December 2024. *Prev Med Rep*. 2025;56:103150. | Adverse events |
| 2. | Domnich A, Orsi A, Lai PL, et al. Enhanced safety surveillance of the adjuvanted respiratory syncytial virus vaccine among Italian older adults. *Vaccine: X*. 2025 Apr 26:100647. | Adverse events |
| 3. | Stowe J, Watson C, Ramsay M, et al. Assessing the risk of Guillain-Barré syndrome in older adults after bivalent RSV pre-F vaccination in England. *medRxiv.* 2025 Aug 19:2025-08. | Adverse events |
| 4. | Poupouzas GI, Georgiadis T, Athanasiou N, et al. Respiratory Syncytial Virus (RSV) Infection Among High-risk Adult Groups Hospitalized for Respiratory Infection Across Two Consecutive Seasons in Greece. *Am. J. Respir. Crit. Care Med.* 2025 May 16;211(Abstracts):A6605-. | Conference abstract |
| 5. | Tartof SY, Aliabadi N, Goodwin G, et al. P-604. Preliminary real-world Abrysvo vaccine effectiveness (VE) against Respiratory Syncytial Virus (RSV)-related lower respiratory tract disease (LRTD) hospitalizations and emergency department (ED) visits—Kaiser Permanente of Southern California (KPSC), November 2023-April 2024. *Open Forum Infect Dis.* 2025;12(Suppl 1):ofae631.802. Published 2025 Jan 29. doi:10.1093/ofid/ofae631.802 | Conference abstract |
| 6. | Tartof SY, Aliabadi N, Goodwin G, et al. 165. Real-world Abrysvo Vaccine Effectiveness (VE) against Respiratory Syncytial Virus (RSV)-related Severe Acute Respiratory Infection (ARI) Hospitalizations and Emergency Department (ED) Visits—Kaiser Permanente of Southern California (KPSC), November 2023–April 2024. In *Open Forum Infect. Dis.* 2025 Feb (Vol. 12, No. Supplement_1, pp. ofae631-002). US: Oxford University Press. | Conference abstract |
| 7. | Walsh EE, Woodside J, Perez Marc G, et al. P-600. Efficacy of a Bivalent RSVpreF Vaccine in Older Adults Across a Second RSV Season. *Open Forum Infect Dis.* 2025;12(Suppl 1):ofae631.798. Published 2025 Jan 29. doi:10.1093/ofid/ofae631.798 | Conference abstract |
| 8. | Ahuva A, Reiko S, Erica C, et al. P-603. Potential Public Health Impact of Respiratory Syncytial Virus (RSV) Vaccines for Prevention of RSV Among Older Adults in the United States, *Open Forum Infect. Dis.,* Volume 12, Issue Supplement_1, February 2025, ofae631.801. | Conference abstract |
| 9. | Hamid O, Mohammed SS, Awadalla M, et al. S1048 Respiratory Syncytial Virus Vaccine is Associated With Better Outcomes in Inflammatory Bowel Disease Patients over 60 Years Old: A US Propensity-Matched Study. *Am. J. Gastroenterol.* 2024 Oct 1;119(10S):S735-6. | Conference abstract |
| 10. | Ison MG, Papi A, Langley JM, et al. 1936. Efficacy of One Dose of the Respiratory Syncytial Virus (RSV) Prefusion F Protein Vaccine (RSVPreF3 OA) in Adults ≥ 60 Years of Age Persists for 2 RSV Seasons*. Open Forum Infect Dis.* 2023;10(Suppl 2):ofad500.2467. Published 2023 Nov 27. doi:10.1093/ofid/ofad500.2467 | Conference abstract |
| 11. | El Mouaddin N, Uhart M, Abily L, et al. EPH158 Assessing the Public Health Benefit of a mRNA-Based Respiratory Syncytial Virus Vaccine (mRNA-1345) Among Adults≥ 65 Years in France. *Value in Health*. 2024 Dec 1;27(12):S250. | Conference abstract |
| 12. | Gennattasio A. “Adjuvanted Vaccine to Prevent Respiratory Syncytial Virus in Adults Ages 60 Years and Older.” *Nurs. Womens Health.* vol. 28,3 (2024): 242-246. doi:10.1016/j.nwh.2024.01.005 | Ineligible study design |
| 13. | Postma, M. J., Cheng, C. Y., Buyukkaramikli, N. C., et al. Predicted Public Health and Economic Impact of Respiratory Syncytial Virus Vaccination with Variable Duration of Protection for Adults ≥60 Years in Belgium. *Vaccines (Basel).* 2023 May 16;11(5):990. doi: 10.3390/vaccines11050990. | Ineligible study design |
| 14. | Mensah AA, Whitaker H, Andrews NJ, Watson CH. Early impact of RSV vaccination in older adults in England. *The Lancet.* 2025 Apr 5;405(10485):1139-40. | Ineligible study design |
| 15. | Lassen MC, Johansen ND, Christensen SH, et al. Bivalent RSV prefusion F protein–based vaccine for preventing cardiovascular hospitalizations in older adults: a prespecified analysis of the DAN-RSV trial. *JAMA.* 2025 Aug 30. | Ineligible study design |
| 16. | Pareek M, Lassen MC, Johansen ND, et al.. Effectiveness of bivalent respiratory syncytial virus prefusion F protein-based vaccine in individuals with or without atherosclerotic cardiovascular disease: the DAN-RSV trial. *Eur. Heart J.* 2025 Aug 30:ehaf679. | Ineligible study design |
| 17. | Tartof SY, Aliabadi N, Goodwin G, et al. Estimated Vaccine Effectiveness for Respiratory Syncytial Virus-Related Lower Respiratory Tract Disease. *JAMA Netw Open*. 024;7(12):e2450832. Published 2024 Dec 2. doi:10.1001/jamanetworkopen.2024.50832 | Overlapping population |
| 18 | Bajema KL, Yan L, Li Y, et al. Respiratory syncytial virus vaccine effectiveness among US veterans, September, 2023 to March, 2024: a target trial emulation study. The Lancet Infectious Diseases 2025; 25:625–633. | Overlapping population |

Overlapping population: The participant populations overlapped with that of a more recent study included in analysis; Ineligible study design: This refers to studies whose design did not meet the study objectives or analytical framework. These included descriptive reports (e.g., clinical reviews), economic evaluations, population-level regression discontinuity analysis, and randomised controlled trials.

### **Table S4:** Quality assessment of included cohort studies using the JBI critical appraisal tool.

| Study | 1 | 2 | 3 | 4 | 5 | 6 | 7 | 8 | 9 | 10 | 11 | Total | Total (%) | RoB |
| --- | --- | --- | --- | --- | --- | --- | --- | --- | --- | --- | --- | --- | --- | --- |
| Bajema et al., 2025 | Yes | Yes | Yes | Yes | Yes | Yes | Yes | Yes | Yes | Yes | Yes | 11 | 100% | Low |
| Godonou et al., 2025 | Yes | Yes | Yes | Yes | Yes | Yes | Yes | Yes | Yes | Yes | Yes | 11 | 100% | Low |

RoB, risk of bias; NA, not applicable.

**Checklist:**

1. Were the two groups similar and recruited from the same population?

2. Were the exposures measured similarly to assign people to both exposed and unexposed groups?

3. Was the exposure measured in a valid and reliable way?

4. Were confounding factors identified?

5. Were strategies to deal with confounding factors stated?

6. Were the groups/participants free of the outcome at the start of the study (or at the moment of exposure)?

7. Were the outcomes measured in a valid and reliable way?

8. Was the follow up time reported and sufficiently long for outcomes to occur?

9. Was follow up complete, and if not, were the reasons to lose to follow up described and explored?

10. Were strategies to address incomplete follow up utilised?

11. Was appropriate statistical analysis used?

### **Table S5:** Quality assessment of included case-control studies using the JBI critical appraisal tool.

| Study | 1 | 2 | 3 | 4 | 5 | 6 | 7 | 8 | 9 | 10 | Total | Total (%) | RoB |
| --- | --- | --- | --- | --- | --- | --- | --- | --- | --- | --- | --- | --- | --- |
| Fry et al., 2025 | Yes | Unclear | Yes | Yes | Yes | Yes | Yes | Yes | Yes | Yes | 9 | 90% | Low |
| Payne et al., 2024 | Yes | Yes | Yes | Yes | Yes | Yes | Yes | Yes | Yes | Yes | 10 | 100% | Low |
| Surie et., 2024 | Yes | Yes | Yes | Yes | Yes | Yes | Yes | Yes | Unclear | Yes | 9 | 90% | Low |
| Surie et al., 2025 | Yes | Yes | Yes | Yes | Yes | Yes | Yes | Yes | Unclear | Yes | 9 | 90% | Low |
| Symes et al., 2025 | Yes | Yes | Yes | Yes | Yes | Yes | Yes | Yes | Yes | Yes | 10 | 100% | Low |
| Tartof et al., 2025 | Yes | Unclear | Yes | Yes | Yes | Yes | Yes | Yes | Unclear | Yes | 8 | 80% | Low |

RoB, risk of bias.

**Checklist:**

1. Were the groups comparable other than presence of disease in cases or absence of disease in controls?

2. Were cases and controls matched appropriately?

3. Were the same criteria used for identification of cases and controls?

4. Was exposure measured in a standard, valid and reliable way?

5. Was exposure measured in the same way for cases and controls?

6. Were confounding factors identified?

7. Were strategies to deal with confounding factors stated?

8. Were outcomes assessed in a standard, valid and reliable way for cases and controls?

9. Was the exposure period of interest long enough to be meaningful?

10. Was appropriate statistical analysis used?

### **Table S6.** Sensitivity analyses for main outcomes.

| Outcomes | Analysis | N | Mandel–Paule method | | | DerSimonian and Laird method | | |
| --- | --- | --- | --- | --- | --- | --- | --- | --- |
|  |  |  | **OR (95%CI)** | **I^2^** | 𝜏**^2^** | **OR (95%CI)** | **I^2^** | 𝜏**^2^** |
| RSV-related hospitalisations | Primary analysis | 6 | 0.23 (0.20, 0.27) | 6.5% | 0.002 | 0.23 (0.20, 0.27) | 6.5% | 0.002 |
|  | **Sensitivity analysis** |  |  |  |  |  |  |  |
|  | Excluding cohort studies | 5 | 0.24 (0.19, 0.29) | 11.3% | 0.005 | 0.24 (0.19, 0.29) | 11.3% | 0.004 |
|  | Excluding outlier studies | 5 | 0.23 (0.19, 0.28) | 11.7% | 0.004 | 0.23 (0.20, 0.28) | 11.7% | 0.004 |
|  | Substituting single-season study with two-season study* | 6 | 0.24 (0.21, 0.26) | 0.0% | <0.001 | 0.24 (0.21, 0.26) | 0.0% | <0.001 |
| RSV-related ED visits | Primary analysis | 4 | 0.23 (0.21, 0.27) | 0.0% | <0.001 | 0.23 (0.21, 0.27) | 0.0% | <0.001 |
|  | **Sensitivity analysis** |  |  |  |  |  |  |  |
|  | Excluding cohort studies | 3 | 0.24 (0.20, 0.28) | 0.0% | <0.001 | 0.24 (0.20, 0.28) | 0.0% | <0.001 |
|  | Excluding outlier studies | 3 | 0.24 (0.20, 0.28) | 0.0% | <0.001 | 0.24 (0.20, 0.28) | 0.0% | <0.001 |

ED, emergency department; N, number of studies. *Surie et al., (2025) included participants from two RSV seasons: 2023–24 (~83%) and 2024–25 (~17%). Hence, earlier study restricted to the 2023–24 RSV season was replaced with its updated version including participants from both the 2023–24 and 2024–25 seasons to assess the impact on pooled estimates.

###

### **Table S7.** Sensitivity analysis of specific subgroups for RSV-related hospitalisations.

| Subgroups | Analysis | N | Mandel–Paule method | | | DerSimonian and Laird method | | |
| --- | --- | --- | --- | --- | --- | --- | --- | --- |
|  |  |  | **OR (95%CI)** | **I^2^** | 𝜏**^2^** | **OR (95%CI)** | **I^2^** | 𝜏**^2^** |
| Immunocompromised | Primary analysis | 4 | 0.31 (0.27, 0.34) | 0.0% | <0.001 | 0.31 (0.27, 0.34) | 0.0% | <0.001 |
|  | **Sensitivity Analysis** |  |  |  |  |  |  |  |
|  | Excluding outlier studies | 3 | 0.31 (0.25, 0.37) | 0.0% | <0.001 | 0.31 (0.25, 0.37) | 0.0% | <0.001 |
| Age 60-74 years | Primary analysis | 3 | 0.27 (0.10, 0.71) | 55.09% | 0.099 | 0.27 (0.10, 0.67) | 55.9% | 0.063 |
|  | **Sensitivity Analysis** |  |  |  |  |  |  |  |
|  | Excluding outlier studies | 2 | 0.24 (0.11, 0.56) | 0.0% | <0.001 | 0.24 (0.11,0.56) | 0.0% | <0.001 |
| RSVpreF, Abrysvo | Primary analysis | 4 | 0.23 (0.13, 0.41) | 2.7% | 0.003 | 0.23 (0.13, 0.41) | 2.7% | 0.004 |
|  | **Sensitivity Analysis** |  |  |  |  |  |  |  |
|  | Excluding outlier studies | 3 | 0.24 (0.11, 0.53) | 12.3% | 0.012 | 0.24 (0.11, 0.53) | 12.3% | 0.013 |

N, number of studies.

Sensitivity analyses by subgroups also supported the main findings for RSV-related hospitalisation (appendix p 15). Among immunocompromised participants, the pooled OR remained unchanged after excluding outlier studies with high SEs (OR: 0.31; 95% CI: 0.25–0.37), with also low heterogeneity observed in the sensitivity analysis (I² = 0·0%). After adjusting for outliers, the Kruskal-Wallis test showed a significant difference between subgroups by immune status (p = 0.049), consistent with the results of the primary analysis.

For older adults aged 60–74 years, excluding outlier studies yielded a slightly lower odds of RSV-related hospitalisation (OR: 0.24; 95% CI: 0.11–0.56; I² = 0.0%), though with a narrow confidence interval, compared with the primary analysis for this subgroup. After this adjustment, there was no significant difference in effect estimates across age groups (Kruskal–Wallis test, p = 0.767).

For RSVpreF vaccine type, exclusion of outlier studies produced a similar odds of RSV-related hospitalisation (OR: 0.24; 95% CI: 0.11–0.53; I² = 12·3%) comparable to that of the primary analysis, with no statistically significant differences observed (Kruskal-Wallis test, p = 0.248).

### **Table S8.** The overall certainty of RSV-related hospitalisations and ED visits using GRADE. **Population:** Older adults (≥ 60 years); **Interventions:** US-FDA approved RSV vaccines (Abrysvo, Arexvy); **Comparison:** No interventions (unvaccinated); **Outcomes:** Vaccine effectiveness (VE) against RSV-related hospitalisation and ED visits; **Setting:** Real-world clinical practice.

| **Certainty assessment** | | | | | | | **No of patients** | | **Effect** | | **Certainty** | **Importance** |
| --- | --- | --- | --- | --- | --- | --- | --- | --- | --- | --- | --- | --- |
| **No of studies** | **Study design** | **Risk of bias** | **Inconsistency** | **Indirectness** | **Imprecision** | **Other considerations** | **Vaccines** | **Control** | **Relative  (95% CI)** | **Absolute  (95% CI)** |  |  |
| **RSV-related hospitalisations** | | | | | | | | | | | | |
| 6 | Observational studies | Not serious | Not serious | Not serious | Not serious | None | 318214 | 641154 | VE 77% (73% to 80%); RR 0.23 (0.20 to 0.27) | - | ⨁⨁⨁◯  Moderate^a^ | Critical^1^ |
| **RSV-related ED visits** | | | | | | | | | | | | |
| 4 | Observational studies | Not serious | Not serious | Not serious | Not serious | None | 310692 | 559720 | VE 77% (73% to 79%); RR 0.23 (0.21 to 0.27) | - | ⨁⨁⨁◯  Moderate^a^ | Important^1^ |

**RSV:** respiratory syncytial virus; **ED:** emergency department; **CI:** confidence interval; **VE:** vaccine effectiveness; **RR:** risk ratio

#### **Explanations**

#### ^a^ Certainty was rated as moderate because all studies were observational but upgraded by one level for a large effect size (RR <0.5), based on direct evidence with no serious risk of bias or imprecision. Event rates were rare (<10%), so odds ratios approximate risk ratios; GRADE thresholds for large (RR >2 or <0.5) and very large effects (RR >5 or <0.2) were applied directly to OR values, consistent with current guidance [10,11].

#### ^1^ Outcome importance was rated according to GRADE guidance: hospitalisation as critical, ED visits as important for decision-making.

### **Table S9.** Narrative synthesis of vaccine effectiveness estimates against incidence of RSV‑related LRTI and ICU admission from included studies.

| **Study** | **Country** | **Study design** | **Outcome** | **Case definition** | **Timeframe** | **VE (95% CI)** | **Adjusted covariates** |
| --- | --- | --- | --- | --- | --- | --- | --- |
| I**ncidence of RSV-related LRTI** | | | | | | | |
| Bajema et al., 2025 | US | Retrospective cohort | Documented RSV infection | Clinical and lab confirmed RSV | September 2023 to March 2024 | 77.47  (73.20, 81.42) | Age, sex, race, ethnicity, underlying conditions, and geographic location |
| Fry et al., 2025 | US | Test-negative case-control | MA RSV illness | Clinical and lab confirmed RSV | October 2023 to April 2024 | 75.1  (73.6, 76.4) | Age, month of RSV test, immunocompromised status, and state of residence |
| Godonou et al., 2025 | US | Prospective cohort | Lab-confirmed RSV infection | PCR positive | August 2023 to March 2024 | 50.8  (-79.1, 86.5) | Sex, race, ethnicity, and presence of high-risk health conditions |
| **ICU admission** | | | | | | | |
| Bajema et al., 2025 | US | Retrospective cohort | ICU admission | Clinical and lab confirmed RSV | September 2023 to March 2024 | 87.15  (61.98, 100.00) | Age, sex, race, ethnicity, underlying conditions, and geographic location |

RSV: respiratory syncytial virus; VE: vaccine effectiveness; CI: confidence interval; LRTI: lower respiratory tract infection; MA: medically attended; PCR: polymerase chain reaction; ICU, intensive care unit.

# **SUPPLEMENTARY FIGURES**

### **Figure S1.** Subgroup analysis of RSV-related hospitalisation by age, comparing RSV vaccinated individuals with controls.


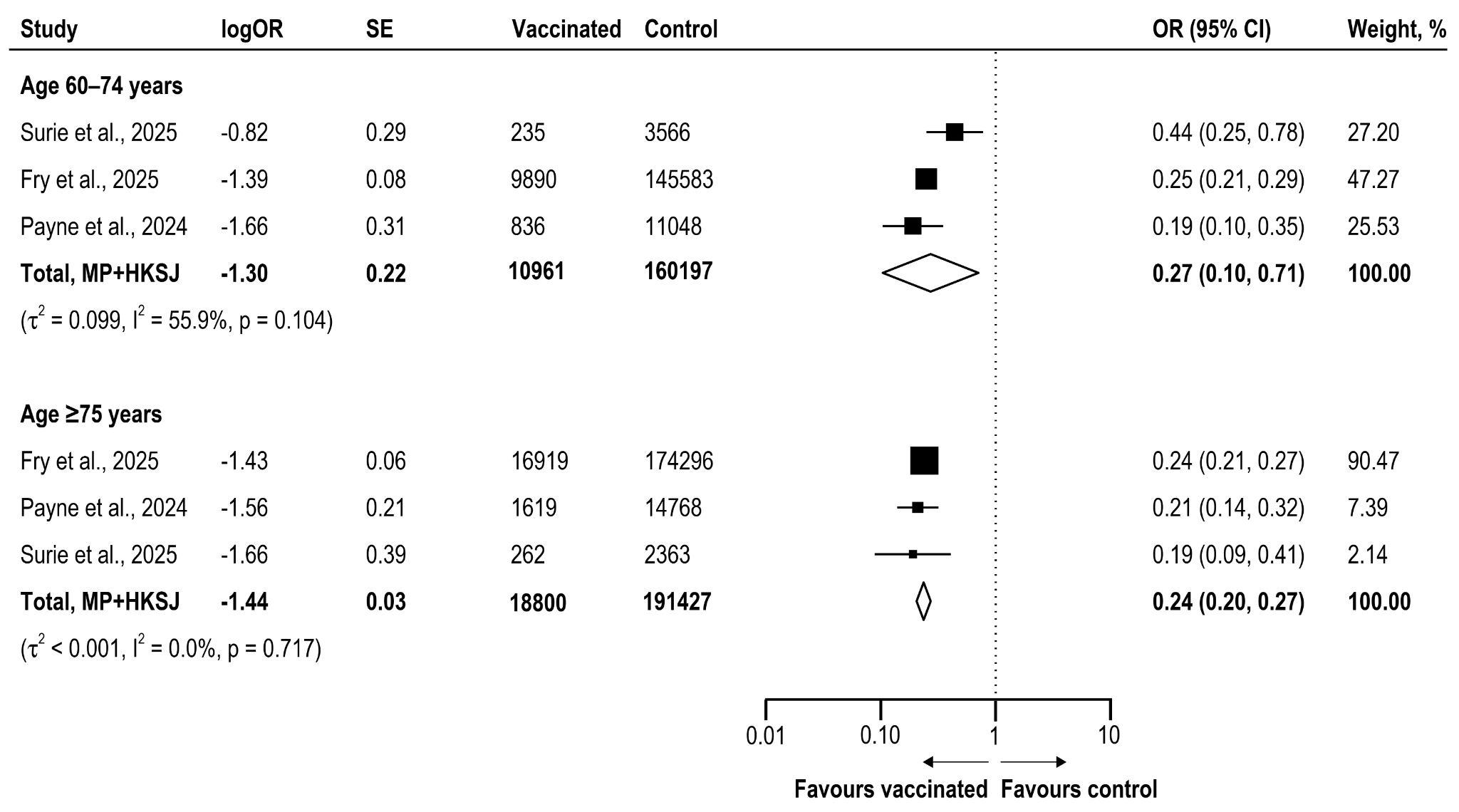


**Figure S2.** Subgroup analysis of RSV-related hospitalisation by vaccine type, comparing RSV vaccinated individuals with controls.


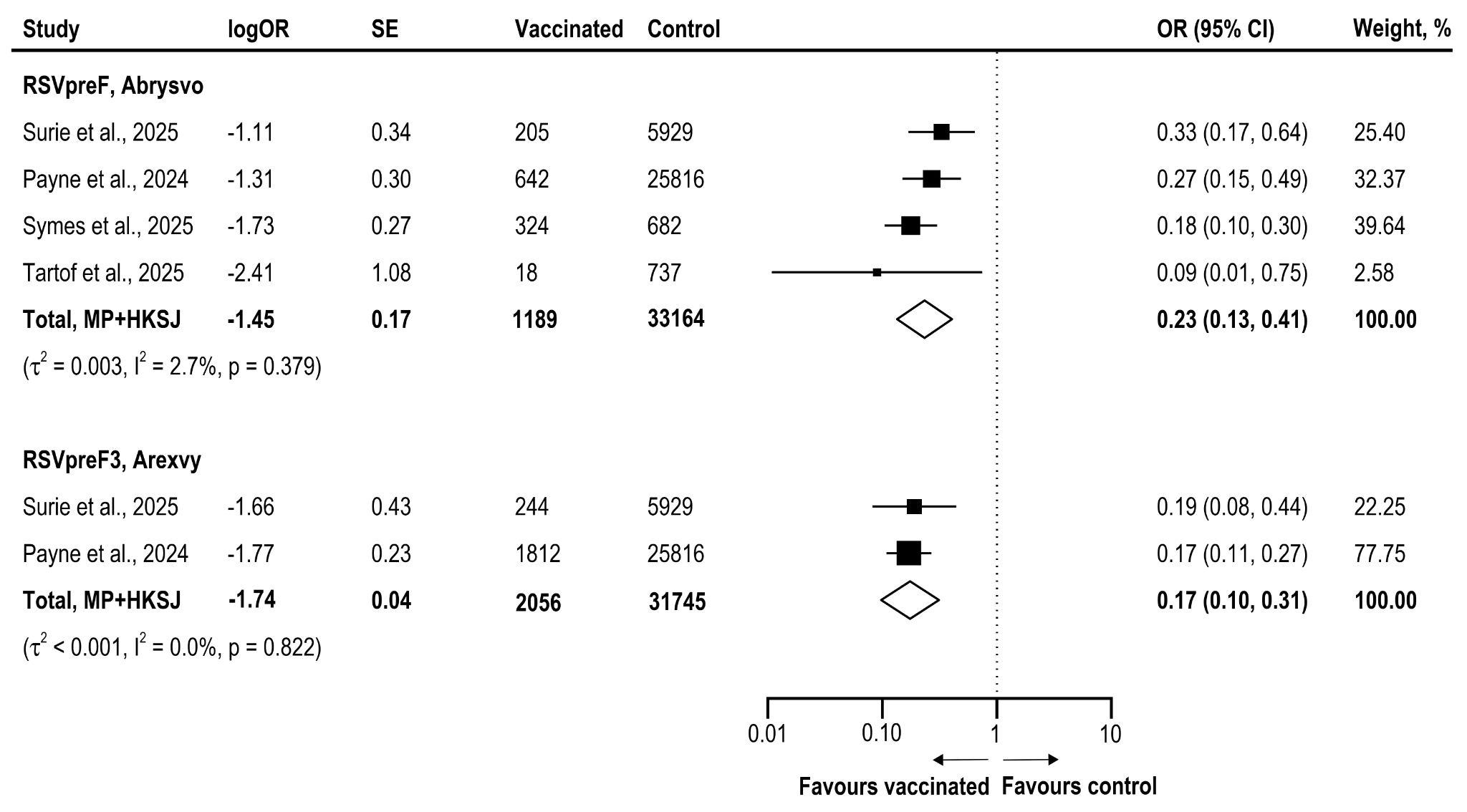


**Figure S3.** Bubble plot from meta-regression examining the association between study duration (in days) and RSV-related hospitalisations. Bubble size reflects the relative weight of each study in the analysis.


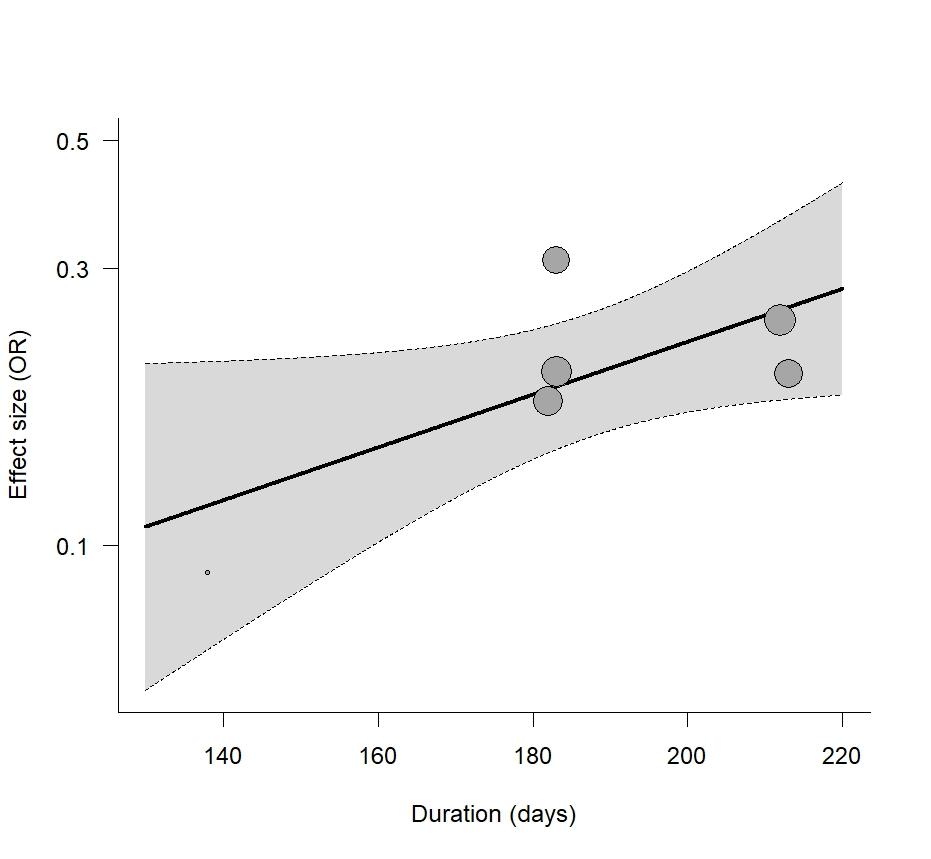


### **Figure S4.** Bubble plot from meta-regression examining the association between case definition stringency and RSV-related hospitalisations. Bubble size reflects the relative weight of each study in the analysis.


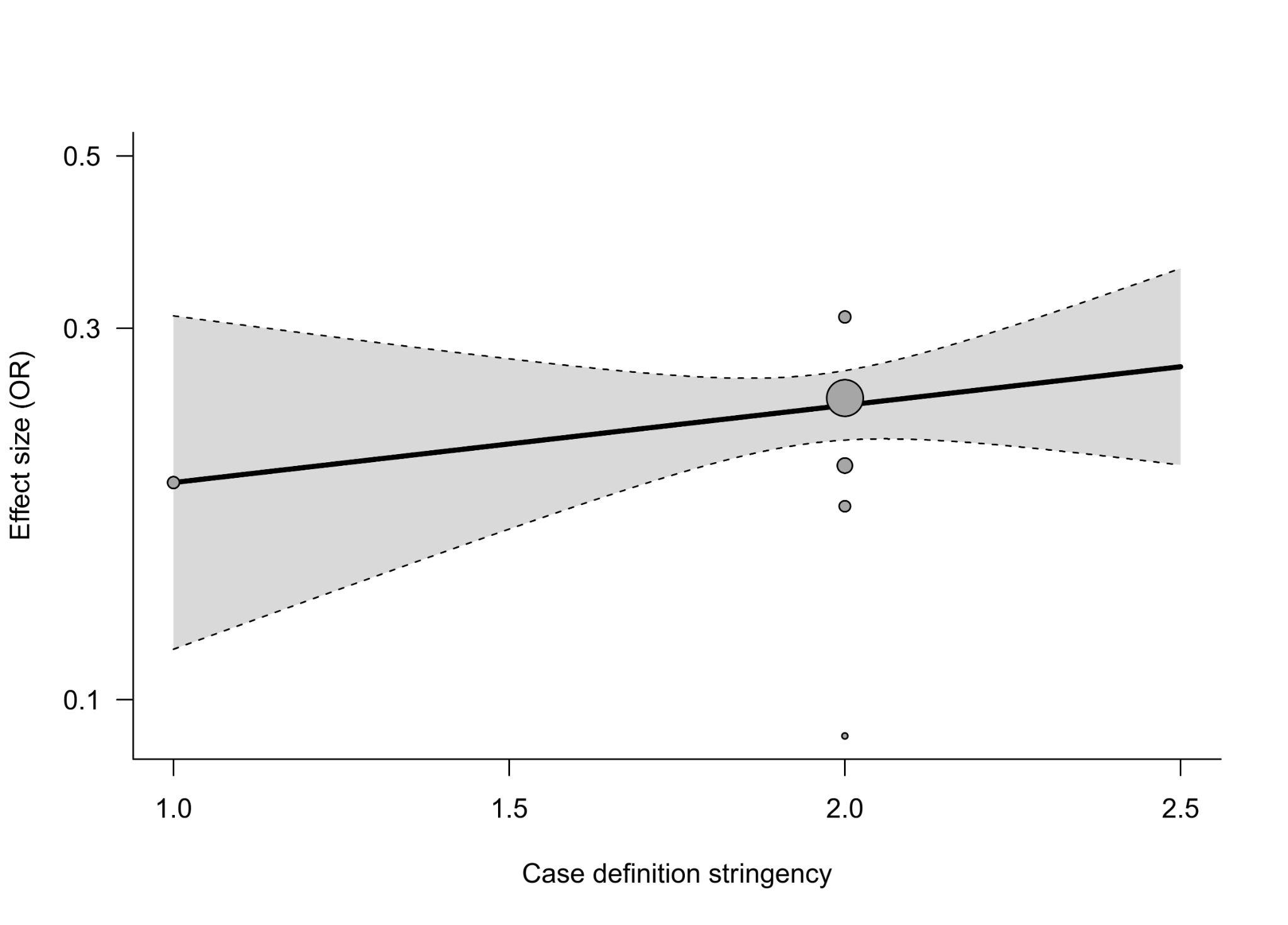


### **Figure S5.** Funnel plot of studies reporting RSV-related hospitalisations for older adults aged 60 years or older. SE: standard error.


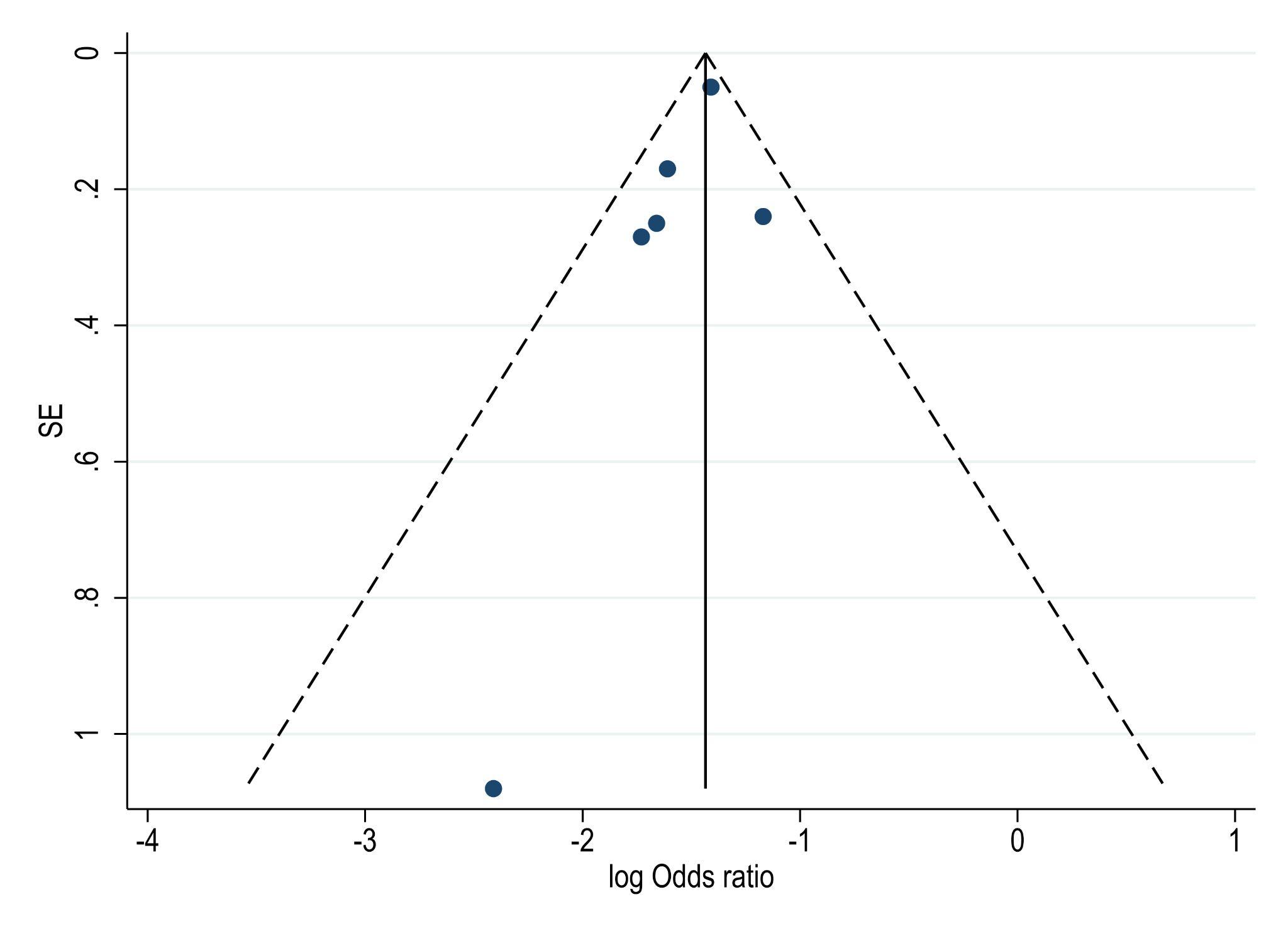


### **Figure S6.** Funnel plot of studies reporting RSV-related emergency department visits for older adults aged 60 years or older. SE: standard error.


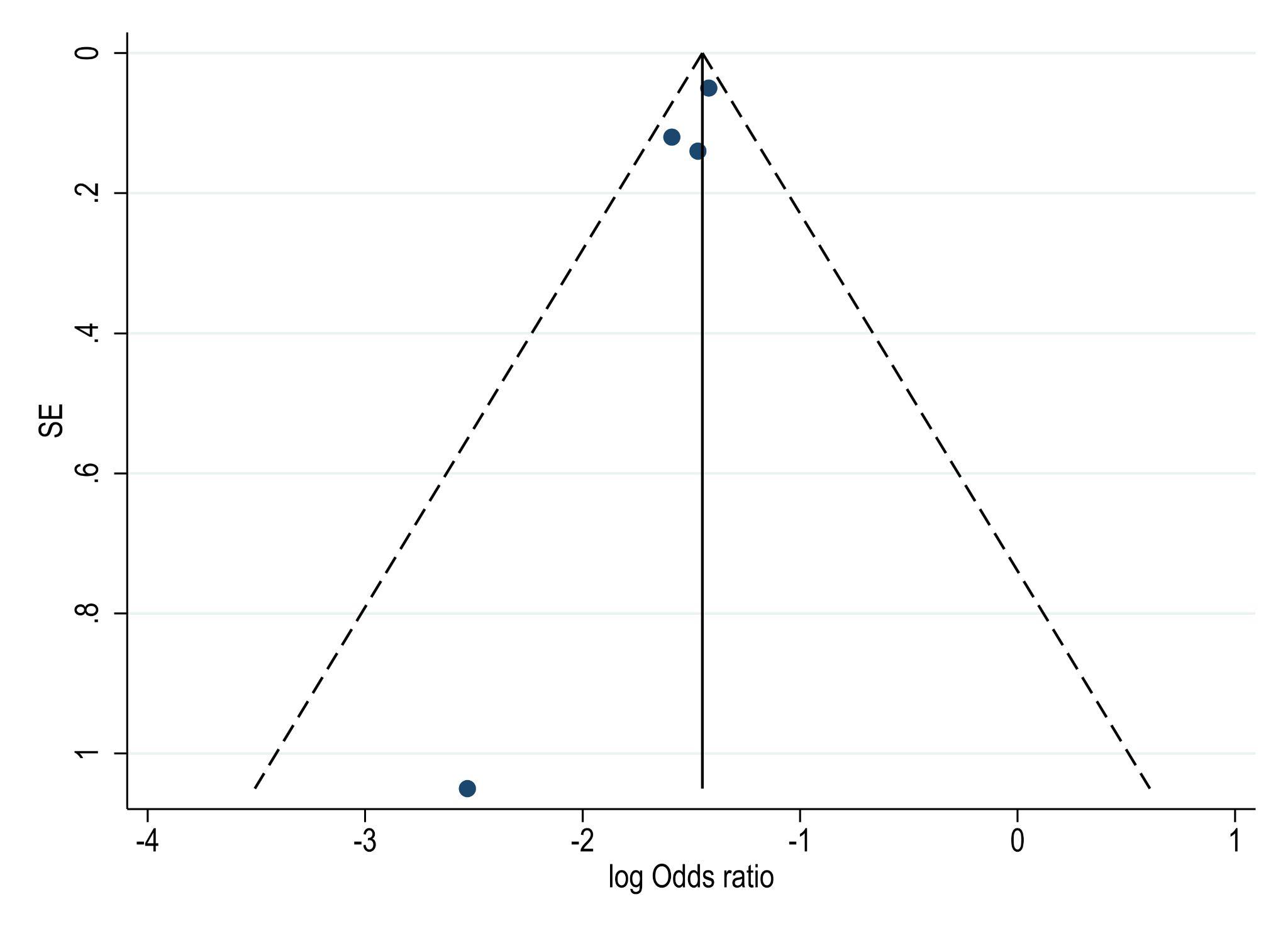


# **References**

- - - 1. Hill JA, Boonyaratanakornkit J, Mikulska M, et al. Innovation in active and passive immunisation of people who are immunocompromised: a call to action. The Lancet Infectious Diseases **2026**; 26:e16–e29.
      2. Bajema KL, Bui DP, Yan L, et al. Durability of Respiratory Syncytial Virus Vaccine Effectiveness Among US Veterans. JAMA Intern Med **2026**; 186:78.
      3. Fry SE, Terebuh P, Kaelber DC, Xu R, Davis PB. Effectiveness and Safety of Respiratory Syncytial Virus Vaccine for US Adults Aged 60 Years or Older. JAMA Netw Open **2025**; 8:e258322.
      4. Payne AB, Watts JA, Mitchell PK, et al. Respiratory syncytial virus (RSV) vaccine effectiveness against RSV-associated hospitalisations and emergency department encounters among adults aged 60 years and older in the USA, October, 2023, to March, 2024: a test-negative design analysis. The Lancet **2024**; 404:1547–1559.
      5. Surie D, Self WH, Yuengling KA, et al. RSV Vaccine Effectiveness Against Hospitalization Among US Adults Aged 60 Years or Older During 2 Seasons. JAMA **2025**; Available at: https://jamanetwork.com/journals/jama/fullarticle/2838490. Accessed 23 October 2025.
      6. Symes R, Whitaker HJ, Ahmad S, et al. Vaccine effectiveness of a bivalent respiratory syncytial virus (RSV) pre-F vaccine against RSV-associated hospital admission among adults aged 75–79 years in England: a multicentre, test-negative, case–control study. The Lancet Infectious Diseases **2025**; :S1473309925005468.
      7. Tartof SY, Aliabadi N, Goodwin G, et al. Estimated Vaccine Effectiveness for Respiratory Syncytial Virus–Related Acute Respiratory Illness in Older Adults: Findings From the First Postlicensure Season. Clin Infect Dis **2025**; :ciaf496.
      8. Surie D, Self WH, Zhu Y, et al. RSV Vaccine Effectiveness Against Hospitalization Among US Adults 60 Years and Older. JAMA **2024**; 332:1105–1107.
      9. Godonou E-T, Callear AP, Juntila-Raymond CL, et al. Respiratory syncytial virus (RSV) vaccine effectiveness and antibody correlates of protection among older adults in the Community Vaccine Effectiveness (CoVE) observational study. eBioMedicine **2025**; 121:105961.
      10. Norton EC, Dowd BE, Maciejewski ML. Odds Ratios—Current Best Practice and Use. JAMA **2018**; 320:84.
      11. Cummings P. The Relative Merits of Risk Ratios and Odds Ratios. Arch Pediatr Adolesc Med **2009**; 163:438.
